# Perfusion Measures and Outcomes (PERForm) registry: First annual report

**DOI:** 10.1051/ject/2024006

**Published:** 2024-06-18

**Authors:** David C. Fitzgerald, Xiaoting Wu, Timothy A. Dickinson, Donald Nieter, Erin Harris, Shelby Curtis, Emily Mauntel, Amanda Crosby, Gaetano Paone, Joshua B. Goldberg, Alphonse DeLucia, Kaushik Mandal, Patricia F. Theurer, Carol Ling, Jeffrey Chores, Donald S. Likosky

**Affiliations:** 1 Medical University of South Carolina College of Health Professions 151-A Rutledge Avenue, A321 Charleston SC 29425 USA; 2 Department of Cardiac Surgery, Michigan Medicine, University of Michigan 1500 E Medical Center Dr., 5144 Cardiovascular Center Ann Arbor MI 48109-5864 USA; 3 Division of Cardiovascular Surgery, Mayo Clinic 200 First Street SW Rochester MN 55905 USA; 4 Michigan Society of Thoracic and Cardiovascular Surgeons Quality Collaborative Arbor Lakes Building 3 #3130/4251 Plymouth Road Ann Arbor MI 48105 USA; 5 Department of Perfusion Services, University of Tennessee Medical Center 1924 Alcoa Hwy Knoxville TN 37920 USA; 6 Division of Cardiothoracic Surgery, Emory University School of Medicine 550 Peachtree Street, NE Davis-Fischer Bldg, 4th Floor Atlanta GA 30308 USA; 7 Department of Cardiothoracic Surgery, Weill Cornell Medical Center/New York Presbyterian Hospital 525 E 68th St M 404 New York NY 10065 USA; 8 Department of Cardiac Surgery, University of Michigan Health West 2122 Health Dr. SW, Suite 133 Wyoming MI 49519 USA; 9 Cardiovascular Services, Detroit Medical Center Sinai Grace Hospital 6001 West Outer Drive Suite POB 321 Detroit MI 48235 USA; 10 Cardiovascular Services, Ascension St. John Providence Health System 16001 West Nine Mile Road Southfield MI 48075 USA

**Keywords:** CABG, Surgery – Aortic valve, Replacement – Cardioplegia, Cardiopulmonary bypass – (CPB)

## Abstract

*Background*: The Perfusion Measures and Outcomes (PERForm) registry was established in 2010 to advance cardiopulmonary bypass (CPB) practices and outcomes. The registry is maintained through the Michigan Society of Thoracic and Cardiovascular Surgeons Quality Collaborative and is the official registry of the American Society of Extracorporeal Technology. *Methods*: This first annual PERForm registry report summarizes patient characteristics as well as CPB-related practice patterns in adult (≥18 years of age) patients between 2019 and 2022 from 42 participating hospitals. Data from PERForm are probabilistically matched to institutional surgical registry data. Trends in myocardial protection, glucose, anticoagulation, temperature, anemia (hematocrit), and fluid management are summarized. Additionally, trends in equipment (hardware/disposables) utilization and employed patient safety practices are reported. *Results*: A total of 40,777 adult patients undergoing CPB were matched to institutional surgical registry data from 42 hospitals. Among these patients, 54.9% underwent a CABG procedure, 71.6% were male, and the median (IQR) age was 66.0 [58.0, 73.0] years. Overall, 33.1% of the CPB procedures utilized a roller pump for the arterial pump device, and a perfusion checklist was employed 99.6% of the time. The use of conventional ultrafiltration decreased over the study period (2019 vs. 2022; 27.1% vs. 24.9%) while the median (IQR) last hematocrit on CPB has remained stable [27.0 (24.0, 30.0) vs. 27.0 (24.0, 30.0)]. Pump sucker termination before protamine administration increased over the study period: (54.8% vs. 75.9%). *Conclusion*: Few robust clinical registries exist to collect data regarding the practice of CPB. Although data submitted to the PERForm registry demonstrate overall compliance with published perfusion evidence-based guidelines, noted opportunities to advance patient safety and outcomes remain.

## Introduction

The practice of cardiopulmonary bypass (CPB) has dramatically improved since its advent in the 1950s [1]. New and emerging data have informed evidence-based clinical guidelines and professional society standards and guidelines that contribute to the advancement of the conduct of CPB [2–4]. Despite these advancements, wide variation persists in the adoption of professional practice standards and/or evidence-based practices [5, 6]. Advancements in local quality improvement, research, and health policy benefit from rigorous clinical databases; yet, existing cardiac surgical registries (e.g., The Society of Thoracic Surgeons Adult Cardiac National Database) lack important detail concerning the practice of CPB to meaningfully evaluate its associated impact on patient safety and outcomes [7].

Prior work has identified a relationship between the variation in the adoption of evidence-based techniques and technology and morbidity, mortality, and healthcare expenditures [8, 9]. Reducing unwarranted variation in CPB practices can be achieved by measuring and benchmarking processes of care against professional standards and evidence-based clinical practice guidelines. The Perfusion Measures and Outcomes (PERForm) registry was established in 2010 and is administered through the Michigan Society of Thoracic and Cardiovascular Surgeons Quality Collaborative (MSTCVS-QC). The PERForm registry seeks to develop and disseminate quarterly benchmarking reports to member hospitals regarding the practice of CPB [10]. While initially piloted within the state of Michigan, the PERForm registry has expanded to participants across the United States and was recognized in 2017 as the official registry of the American Society of ExtraCorporeal Technology (AmSECT).

This first annual PERForm registry report summarizes overall and annual trends in patient characteristics as well as CPB-related practice patterns among adult (≥18 years of age) patients undergoing cardiac surgery (isolated coronary artery bypass grafting, CABG; isolated valve; CABG/valve) between 2019 and 2022 from 42 U.S. participating hospitals. The goal of this report is to advance benchmarking information for the cardiac surgical community, including CPB practices, adherence to evidence-based guidelines and professionally based standards and guidelines, and intraoperative adverse events. Findings derived from this report distinctively advance benchmarking activities relative to other traditional mechanisms and underscore the importance of participating in observational clinical registries for quality assessment and improvement.

## Materials and methods

This study was approved by the University of Michigan’s IRB (HUM00164136, “Notice of Not Regulated Determination”, 7/24/2019). Data use agreements restrict the distribution of raw study-related data files. Requests for summary statistics will be reviewed and may be approved by the study team. The centralized IRB governs both the housing and use of all submitted data.

Data used for this study included cardiac surgical operations that required the use of CPB and were performed between January 1, 2019, and December 31, 2022. The present report covers the period starting with PERForm version 4 given this update involved a significant change in registry fields. Data elements (e.g., extracorporeal circuit characteristics, anemia, blood product utilization, myocardial protection, temperature, aortic disease, medications, safety, and duration indices) are submitted through a secure web portal to a dedicated data warehouse developed by a certified STS vendor [10, 11]. Perfusion data are in turn probabilistically matched with the participating center’s Society of Thoracic Surgeons Adult Cardiac Surgery Database (STS-ACSD) using a published algorithm to provide a more comprehensive assessment of operative practices and their associated impact on clinical outcomes. Surgical harvest files were also used to ascertain patient characteristics and estimate the patient’s preoperative risk of major morbidity and mortality. The occurrence of any of the following ten intraoperative adverse events were reported, including arterial air, oxygenator failure, pump head failure, low venous reservoir level, any electrical failure, gas supply failure, thrombus clot in the circuit, airlock, venous air, and others. While the PERForm registry tracks manufacturer-specific equipment (including disposables), this report only covers the manufacturer associated with specific perfusion electronic medical record systems.

Continuous variables are presented as the median (interquartile range), while categorical variables are presented as counts and percentages. Comparisons across surgical years (2019–2022) were made using both Pearson’s Chi-Square and Fisher’s Exact tests for categorical variables, and Student’s *t*-tests and Wilcoxon rank-sum tests for continuous variables. The degree of missingness across variables is reported. A *p-*value less than 0.05 was considered for all two-tailed significance testing. Statistical analyses were conducted using SAS version 9.4 (SAS Institute, Cary, NC), R version 4.3.1 (R Foundation for Statistical Computing, Vienna, Austria) and RStudio version 2023.6.2.561 (Posit Software, PBC, Boston, MA) [11–13].

## Results

**Figure 1:**
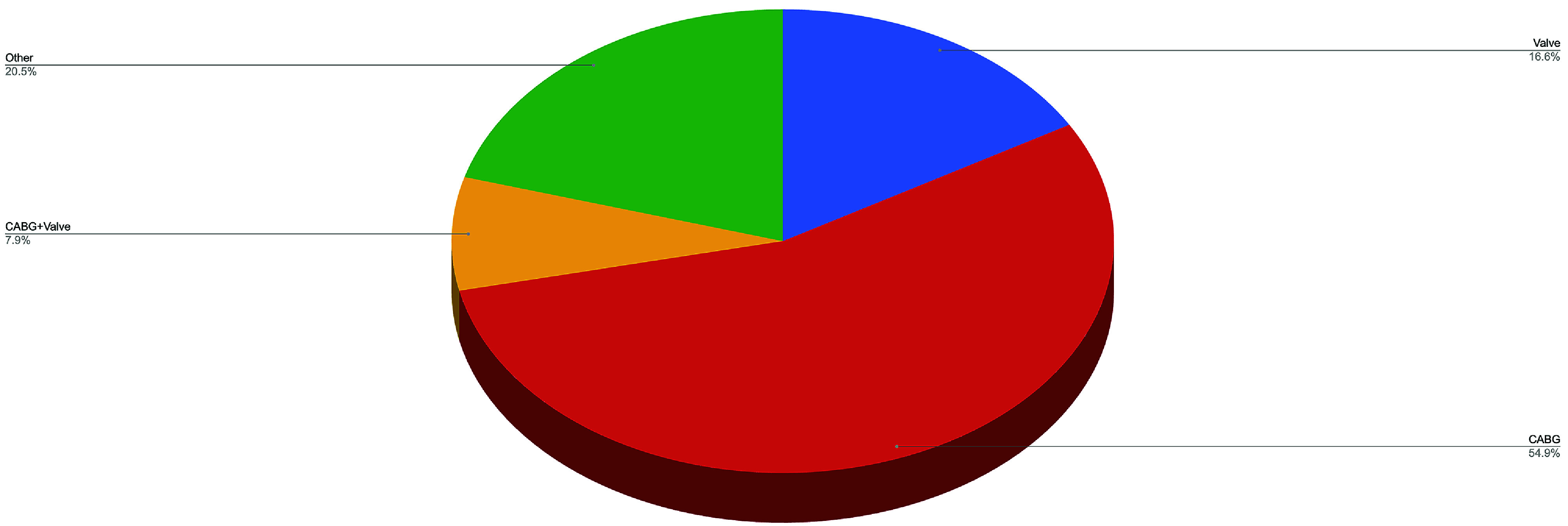
Procedure types submitted to the PERForm registry between 2019 and 2022. Categorical variables are expressed as count (%). Valve procedures include aortic, mitral, tricuspid, and pulmonic valves. Abbreviation: coronary artery bypass grafting (CABG).

**Figure 2:**
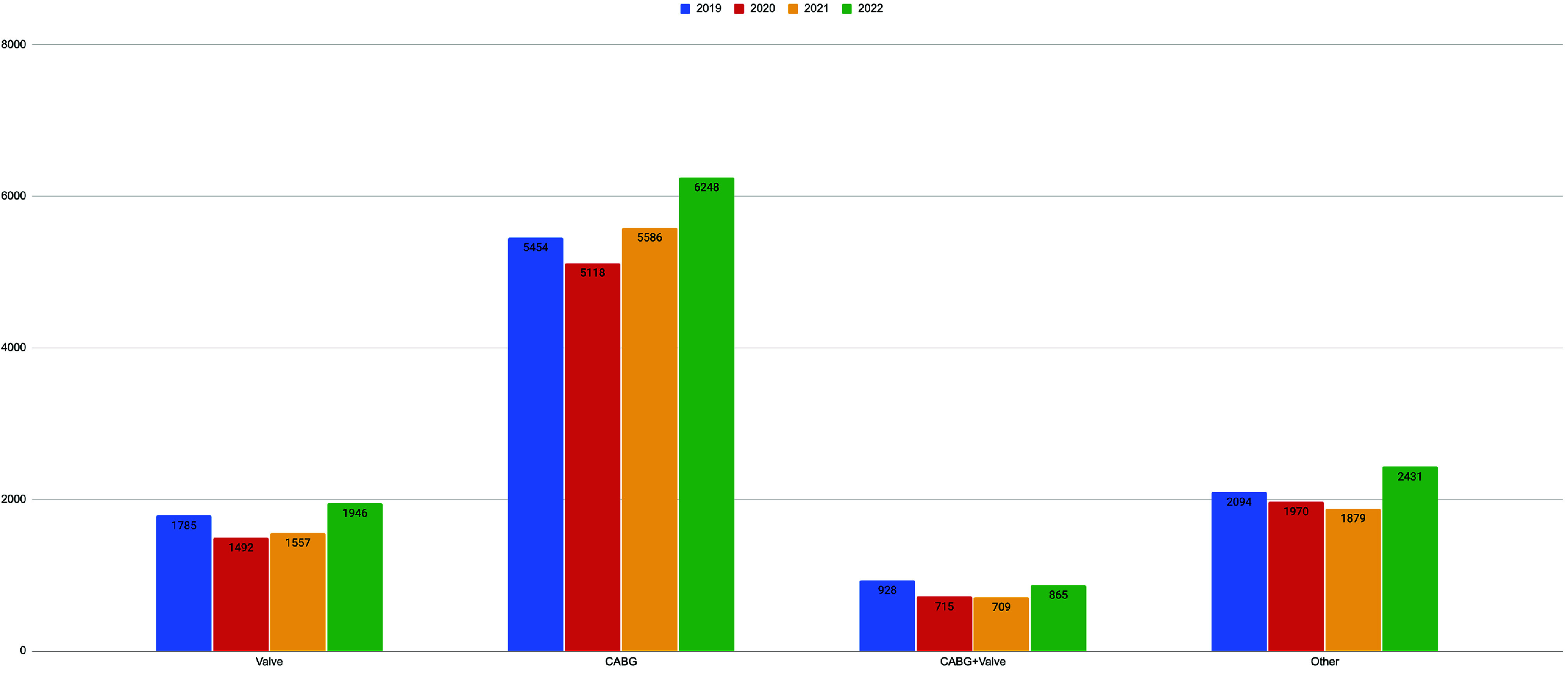
Trends in procedure types between 2019 and 2022. Categorical variables are expressed as counts. Valve procedures include aortic, mitral, tricuspid, and pulmonic valves. Abbreviation: coronary artery bypass grafting (CABG).

A total of 40,777 adult patients underwent cardiac surgery requiring CPB support (Table 1). The percentage of procedures utilizing CPB support did not change over time (2019: 77%, 2022: 77%). The surgical case volume decreased by 13.6% between 2019 and 2020 (10,261 vs. 9295). With the addition of 4 contributing hospitals to the Registry, case volume increased 23.6% between 2021 and 2022 (9731 vs. 11,490). The median (IQR) number of procedures per hospitals was 181.5 (121.8–277.2), a figure that was qualitatively consistent over time. Patients were more commonly male (71.6%), Caucasian (80.7%), and without a history of prior cardiac surgery (92.6%). Major morbidities (stroke/cerebrovascular accident, surgical re-exploration, deep sternal wound infection, postoperative renal failure) defined by the STS-ACSD occurred among 34.1% of patients. The most frequently performed procedure was isolated CABG (54.9%), followed by other (22.6%), isolated valve (16.6%), and CABG/valve (7.9%), Figure 1. The annual trends in procedure-specific volume are presented in Figure 2. Additional procedural data using STS-reported categorizations are provided in Supplementary Table 1.

**Table 1:** Procedure type, patient demographics, and major morbidity.

	Overall	Year				*p*-value	Missing (%)
		2019	2020	2021	2022		
Number of cases	40,777	10,261	9295	9731	11,490		
Number of hospitals	42	38	38	38	42		
Number of cases/center, median (IQR)	181.5 (121.8, 277.2)	197.5 (131.0, 295.5)	168.0 (112.8, 234.2)	178.0 (108.0, 272.0)	194.5 (127.2, 308.0)		
Procedure type						<0.001	
Valve	6780 (16.6)	1785 (17.4)	1492 (16.1)	1557 (16.0)	1946 (16.9)		
PredMM^#^, median (%)	9.6 [6.2, 16.6] (*n* = 6780)	9.6 [6.3, 16.7]	9.7 [6.2, 16.7]	9.6 [6.2, 16.3]	9.5 [6.2, 16.5]		
CABG	22,406 (54.9)	5454 (53.2)	5118 (55.1)	5586 (57.4)	6248 (54.4)		
PredMM^#^, median (%)	8.4 [5.7, 13.3] (*n* = 22,406)	8.5 [5.8, 13.7]	8.5 [5.8, 13.5]	8.3 [5.6, 13.4]	8.2 [5.6, 12.9]		
CABG+Valve	3217 (7.9)	928 (9.0)	715 (7.7)	709 (7.3)	865 (7.5)		
PredMM^#^, median (%)	18.1 [12.9, 26.8] (*n* = 3217)	17.9 [12.9, 26.1]	18.9 [13.3, 29.2]	17.7 [12.8, 26.5]	18.1 [12.5, 26.4]		
Other	8374 (20.5)	2094 (20.4)	1970 (21.2)	1879 (19.3)	2431 (21.2)		
PredMM^#^, median (%)	4.8 [3.8, 5.9] (*n* = 8374)	3.4 [3.4, 3.4]	6.2 [4.3, 7.4]	4.8 [4.8, 4.8]	4.80 [3.9, 5.7]		
Demographics							
Gender						0.66	
Male	29,198 (71.6)	7361 (71.7)	6692 (72.0)	6961 (71.5)	8184 (71.2)		
Female	11,579 (28.4)	2900 (28.3)	2603 (28.0)	2770 (28.5)	3306 (28.8)		
Unknown	0 (0.0)	0 (0.0)	0 (0.0)	0 (0.0)	0 (0.0)		
Age, median	66.0 [58.0, 73.0]	66.0 [58.0, 72.0]	66.0 [58.0, 73.0]	66.0 [58.0, 73.0]	66.0 [58.0, 73.0]	0.50	0.5
Race						<0.001	0
Caucasian	32,902 (80.7)	8053 (78.5)	7409 (79.7)	8091 (83.1)	9349 (81.4)		
Black	3505 (8.6)	982 (9.6)	763 (8.2)	809 (8.3)	951 (8.3)		
Other	2202 (5.4)	475 (4.6)	436 (4.7)	550 (5.7)	741 (6.4)		
Unknown	2168 (5.3)	751 (7.3)	687 (7.4)	281 (2.9)	449 (3.9)		
Risk factors							
Diabetes	15,962 (39.1)	3945 (38.4)	3618 (38.9)	3984 (40.9)	4415 (38.4)	<0.001	0
Peripheral arterial disease	5072 (12.4)	1349 (13.1)	1209 (13.0)	1152 (11.8)	1362 (11.9)	0.003	0
Cerebrovascular disease	9336 (22.9)	2402 (23.4)	2208 (23.8)	2214 (22.8)	2512 (21.9)	0.006	0
Chronic lung disease moderate/severe	4391 (10.8)	1044 (10.2)	950 (10.2)	1136 (11.7)	1261 (11.0)	0.002	0.1
White blood cell count						0.003	0
<4.5	2014 (4.9)	505 (4.9)	412 (4.4)	482 (5.0)	615 (5.4)		
4.5–10	32,268 (79.1)	8104 (79.0)	7306 (78.6)	7760 (79.7)	9098 (79.2)		
>10	6495 (15.9)	1652 (16.1)	1577 (17.0)	1489 (15.3)	1777 (15.5)		
New York Heart Association Class III/IV	6512 (16.0)	1613 (15.7)	1312 (14.1)	1642 (16.9)	1945 (16.9)	<0.001	0
Previous myocardial infarction	16,496 (40.5)	4192 (40.9)	3861 (41.5)	3856 (39.6)	4587 (39.9)	0.025	0
First cardiac surgery	37,741 (92.6)	9471 (92.3)	8582 (92.3)	9060 (93.1)	10,628 (92.5)	0.11	0
Ejection fraction, median	58.0 [49.0, 63.0]	58.0 [48.0, 63.0]	57.5 [48.0, 63.0]	58.0 [50.0, 63.0]	58.0 [50.0, 63.0]	<0.001	1.5
Ejection fraction						<0.001	0
<40	5317 (13.0)	1393 (13.6)	1350 (14.5)	1235 (12.7)	1339 (11.7)		
40–50	4802 (11.8)	1243 (12.1)	1116 (12.0)	1141 (11.7)	1302 (11.3)		
50–60	13,732 (33.7)	3127 (30.5)	3133 (33.7)	3354 (34.5)	4118 (35.8)		
≥60	16,926 (41.5)	4498 (43.8)	3696 (39.8)	4001 (41.1)	4731 (41.2)		
Number of diseased vessel (3 or more)	20,677 (50.7)	5196 (50.6)	4769 (51.3)	5043 (51.8)	5669 (49.3)	0.002	0
Current smoker	7728 (19.0)	1851 (18.0)	1878 (20.2)	1836 (18.9)	2163 (18.8)	0.002	0
Cardiogenic shock	1247 (3.1)	335 (3.3)	283 (3.0)	298 (3.1)	331 (2.9)	0.44	0
Status						<0.001	0
Elective	21,504 (52.7)	5281 (51.5)	4557 (49.0)	5326 (54.7)	6340 (55.2)		
Urgent	17,625 (43.2)	4544 (44.3)	4324 (46.5)	4035 (41.5)	4722 (41.1)		
Emergent/emergent salvage	1648 (4.0)	436 (4.2)	414 (4.5)	370 (3.8)	428 (3.7)		
Morbidity/mortality							
Major morbidity (%)	6240 (34.2)	1508 (33.3)	1553 (36.3)	1418 (33.2)	1761 (34.0)	0.008	0
Operative mortality (%)	1349 (3.3%)	321 (3.1%)	339 (3.6%)	331 (3.4%)	358 (3.1%)	0.12	0

Continuous variables are expressed as median, [IQR], and categorical variables as count (%). PredMM^#^, Predicted Risk of Mortality and Morbidity. Major morbidity (stroke/cerebrovascular accident, surgical re-exploration, deep sternal wound infection, postoperative renal failure, prolonged intubation).

Table 2 describes the disposables and monitoring equipment reported during the study period. The majority of CPB disposable components consisted of biocoated circuits (all but cannula – 91.4%) and centrifugal pumps (66.8%), neither of which appreciably changed over time. More than half (57.1%) of procedures were performed without a perfusion electronic medical record. Procedural use of arterial and venous gas trending increased over time (2019 vs. 2022: 27.7% vs. 39.1%).

**Table 2:** Disposables and monitoring equipment.

	Overall	Year				*p*-value	Missing
		2019	2020	2021	2022		
Number of cases	40,777	10,261	9295	9731	11,490		
Bio coating area, *n* (%)						<0.001	0.1
None	11 (0.0)	10 (0.1)	0 (0.0)	0 (0.0)	1 (0.0)		
All but cannula	37,235 (91.4)	9223 (90.0)	8435 (90.8)	9103 (93.7)	10,474 (91.3)		
Limited components	2702 (6.6)	703 (6.9)	686 (7.4)	614 (6.3)	699 (6.1)		
Tip to tip	776 (1.9)	310 (3.0)	165 (1.8)	2 (0.0)	299 (2.6)		
Arterial pump device, *n* (%)						<0.001	0.1%
Roller pump	13,470 (33.1)	3419 (33.4)	2857 (30.8)	3217 (33.1)	3977 (34.7)		
Centrifugal pump	27,220 (66.8)	6830 (66.6)	6402 (68.9)	6506 (66.9)	7492 (65.2)		
Other	87 (0.2)	12 (0.1)	36 (0.4)	8 (0.1)	31 (0.3)		
Perfusion electronic medical record						<0.001	0.1
No perfusion EMR	23,257 (57.1)	6776 (66.1)	5266 (56.7)	5261 (54.1)	5954 (51.9)		
Epic	3556 (8.7)	1 (0.0)	521 (5.6)	762 (7.8)	2272 (19.8)		
General electric – centricity	4932 (12.1)	1276 (12.5)	1151 (12.4)	1217 (12.5)	1288 (11.2)		
Getinge – metavision	1 (0.0)	0 (0.0)	1 (0.0)	0 (0.0)	0 (0.0)		
LivaNova – connect	3060 (7.5)	1088 (10.6)	982 (10.6)	953 (9.8)	37 (0.3)		
LivaNova – DMS	1 (0.0)	0 (0.0)	1 (0.0)	0 (0.0)	0 (0.0)		
Spectrum medical	3422 (8.4)	668 (6.5)	789 (8.5)	932 (9.6)	1033 (9.0)		
Terumo – TLink	1487 (3.7)	338 (3.3)	386 (4.2)	386 (4.0)	377 (3.3)		
Talis-ACG perfusion	1003 (2.5)	95 (0.9)	189 (2.0)	208 (2.1)	511 (4.5)		
Other	5 (0.0)	4 (0.0)	0 (0.0)	0 (0.0)	1 (0.0)		
Cerebral oximetry device usage						<0.001	0.4%
Yes	26,671 (65.5)	7135 (69.5)	5893 (63.4)	9399 (64.7)	7424 (64.2)		
No	14,026 (34.5)	3126 (30.5)	3402 (36.6)	3432 (35.3)	4066 (35.8)		
Inline blood gas trending device						<0.001	0.2
None	2705 (6.6)	892 (8.7)	607 (6.5)	569 (5.9)	637 (5.6)		
Arterial and venous	13,001 (31.9)	2841 (27.7)	2879 (30.0)	3332 (34.2)	4489 (39.1)		
Arterial only	5511 (13.5)	1461 (14.3)	1333 (14.4)	1321 (13.6)	1396 (12.2)		
Venous only	16,786 (41.2)	4474 (43.7)	3939 (42.4)	3854 (39.6)	4519 (39.3)		
Other	1444 (3.5)	450 (4.4)	388 (4.2)	391 (4.0)	215 (1.9)		

Continuous variables are expressed as median, [IQR], and categorical variables as count (%).

Cardioplegia was used in 96.3% of operations, with intermittent delivery being used in 83.8% of operations, Table 3. The most common cardioplegia category was del Nido (40.1%, followed by microplegia (19.5%) and 4:1 ratio (17.0%). Of note, data concerning the maintenance route was incomplete 26.8% of the time. A terminal warm reperfusate (i.e., Hot Shot) was used among 41.9% of procedures, with “blood only” being the most common constituent.

**Table 3:** Cardioplegia details.

	Overall	Year				*p*-value	Missing
		2019	2020	2021	2022		
Number of cases	40,777	10,261	9295	9731	11,490		
Use of cardioplegia						<0.001	0.1
Yes, cardioplegia	39,211 (96.3)	9832 (95.9)	8901 (95.8)	9392 (96.7)	11,086 (96.7)		
Yes, ventricular fibrillation	36 (0.1)	10 (0.1)	6 (0.1)	4 (0.0)	16 (0.1)		
None	1470 (3.6)	409 (4.0)	382 (4.1)	319 (3.3)	360 (3.1)		
Cardioplegia regime						<0.001	4.2
Continuous	5094 (13.0)	1339 (13.7)	1272 (14.3)	1245 (13.3)	1238 (11.2)		
Intermittent	32,745 (83.8)	8454 (86.3)	7615 (85.7)	8051 (86.3)	8625 (78.0)		
Single dose	1239 (3.2)	0 (0.0)	0 (0.0)	38 (0.4)	1201 (10.9)		
Number of doses, median	3.00 [2.00, 6.00]	3.00 [2.00, 6.00]	4.00 [2.00, 6.00]	3.00 [2.00, 6.00]	3.00 [2.00, 6.00]	<0.001	8.1
Total cardioplegia volume (mL), median	2100.0 [1250.0, 3400.0]	2150.0 [1250.0, 3500.0]	2288.5 [1279.5, 3750.0]	2100.0 [1200.0, 3400.0]	2000.0 [1227.0, 3100.0]	<0.001	5.1
Cardioplegia category						<0.001	3.3
None	395 (1.0)	109 (1.1)	93 (1.0)	92 (1.0)	101 (0.9)		
1:1	1 (0.0)	0 (0.0)	1 (0.0)	0 (0.0)	0 (0.0)		
2:1	4 (0.0)	1 (0.0)	1 (0.0)	1 (0.0)	1 (0.0)		
4:1	6689 (17.0)	1809 (18.3)	1357 (15.2)	1348 (14.3)	2175 (19.5)		
8:1	5592 (14.2)	1572 (15.9)	1431 (16.0)	1324 (14.0)	1265 (11.3)		
Crystalloid	123 (0.3)	43 (0.4)	50 (0.6)	29 (0.3)	1 (0.0)		
Variable	2148 (5.4)	561 (5.7)	516 (5.8)	448 (4.7)	623 (5.6)		
Crystalloid (custodial)	841 (2.1)	337 (3.4)	200 (2.2)	113 (1.2)	191 (1.7)		
Microplegia	7687 (19.5)	1666 (16.9)	2063 (23.0)	2129 (22.5)	1829 (16.4)		
del Nido	15,835 (40.1)	3770 (38.2)	3236 (36.1)	3941 (41.7)	4888 (43.8)		
Other	122 (0.3)	9 (0.1)	8 (0.1)	23 (0.2)	82 (0.7)		
Induction routes						<0.001	5
Antegrade-aortic root	35,281 (91.1)	8617 (88.3)	8019 (90.8)	8520 (92.3)	10,125 (92.8)		
Antegrade-coronary ostium	513 (1.3)	123 (1.3)	124 (1.4)	138 (1.5)	128 (1.2)		
Retrograde	2935 (7.6)	1017 (10.4)	691 (7.8)	575 (6.2)	652 (6.0)		
Maintenance route							
Antegrade-aortic root	18,684 (62.6)	4783 (63.6)	4210 (60.2)	4298 (60.2)	5393 (65.9)	<0.001	26.8
Antegrade-coronary ostium (left, right, or both)	2347 (7.9)	583 (7.7)	527 (7.5)	501 (7.0)	736 (9.0)	<0.001	26.8
Antegrade-bypass graft	2936 (9.8)	650 (8.6)	720 (10.3)	752 (10.5)	814 (9.9)	<0.001	26.8
Retrograde	15,631 (52.4)	4055 (53.9)	3838 (54.9)	3869 (54.2)	3869 (47.2)	<0.001	26.8
Terminal warm reperfusate						<0.001	4.4
No	22,647 (58.1)	5867 (60.1)	4915 (55.5)	5167 (55.5)	6698 (60.5)		
Yes, standard	1203 (3.1)	355 (3.6)	357 (4.0)	330 (3.5)	161 (1.5)		
Yes, Buckberg	124 (0.3)	15 (0.2)	68 (0.8)	27 (0.3)	14 (0.1)		
Yes, blood only	10,312 (26.4)	2406 (24.6)	2503 (28.2)	2729 (29.3)	2674 (24.2)		
Yes, combination	4424 (11.3)	1125 (11.5)	1019 (11.5)	1037 (11.1)	1243 (11.2)		
Yes, microplegia	293 (0.8)	0 (0.0)	0 (0.0)	13 (0.1)	280 (2.5)		

Continuous variables are expressed as median, [IQR], and categorical variables as count (%).

Table 4 describes blood product utilization and fluid management. Approximately 36% of all patients received an allogeneic red blood cell transfusion, with 80.0% of those patients receiving blood within the postoperative period. The median (IQR) last pre-CPB hematocrit and last hematocrit on CPB were 36 (30–39) and 27 (24.0–30.3), respectively. The median (IQR) hematocrit at the time of the first transfusion was 21% (19.0–23.0). Retrograde autologous prime was performed in 85.8% of all procedures, although diminished in use over time (86.5% vs. 83.4%). The median (IQR) indexed net prime volume rose from 321.3 mL/min/m^2^ (278.3–394.9) in 2019 to 349.2 mL/min/m^2^ (288.4–427.9) in 2022. Common prime constituents included heparin, balanced electrolyte solutions, sodium bicarbonate, and mannitol (Supplementary Table 2).

**Table 4:** Blood product utilization and fluid management.

	Overall	Year				*p*-value	Missing
		2019	2020	2021	2022		
Number of cases	40,777	10,261	9295	9731	11,490		
Blood product utilization							
Red blood cell units in the prime (% yes)	1036 (2.5)	261 (2.5)	243 (2.6)	229 (2.4)	303 (2.6)	0.569	0
Red blood cell units in the prime	0.0 [0.0, 0.0]	0.0 [0.0, 0.0]	0.0 [0.0, 0.0]	0.0 [0.0, 0.0]	0.0 [0.0, 0.0]	<0.001	33.3
Red blood cell transfusions						<0.001	0
None	26,084 (64.0)	6825 (66.5)	5862 (63.1)	6168 (63.4)	7229 (62.9)		
Intraoperative only	2929 (7.2)	723 (7.0)	631 (6.8)	728 (7.5)	847 (7.4)		
Postoperative only	7196 (17.6)	1695 (16.5)	1691 (18.2)	1733 (17.8)	2077 (18.1)		
Intraoperative and postoperative	4568 (11.2)	1018 (9.9)	1111 (12.0)	1102 (11.3)	1337 (11.6)		
Hematocrit values, median							
First HCT in room	38.0 [34.0, 42.0]	38.0 [34.0, 41.0]	38.0 [34.0, 42.0]	38.0 [34.0, 42.0]	38.0 [34.0, 42.0]	<0.001	1
Last pre-CPB HCT	35.0 [30.0, 39.0]	34.4 [30.0, 38.1]	34.6 [30.0, 39.0]	34.8 [30.1, 38.9]	35.0 [30.6, 39.0]	<0.001	3.9
First HCT on CPB	27.7 [24.0, 31.1]	27.0 [24.0, 31.0]	27.7 [24.0, 31.5]	27.9 [24.0, 31.2]	28.0 [24.2, 31.2]	<0.001	0.3
Nadir HCT on CPB	26.0 [22.1, 29.2]	26.0 [22.0, 29.0]	26.0 [22.0, 29.7]	26.0 [22.5, 29.4]	26.0 [22.6, 29.2]	<0.001	0.4
Last HCT on CPB	27.0 [24.0, 30.3]	27.0 [24.0, 30.0]	27.0 [24.0, 30.8]	27.0 [24.0, 30.3]	27.0 [24.0, 30.2]	<0.001	0.4
Prior to first intraoperative transfusion	21.0 [19.0, 23.0]	20.0 [19.0, 23.0]	21.0 [19.0, 23.0]	21.0 [19.0, 23.0]	21.0 [19.0, 23.0]	0.01	4.2
Prior to second intraoperative transfusion	21.0 [19.0, 23.0]	21.0 [19.0, 24.0]	21.0 [19.0, 23.0]	21.0 [19.0, 23.0]	21.0 [19.0, 24.0]	0.213	11.8
Fluid management							
Total pre-bypass perioperative crystalloid volume, median	900.0 [600.0, 1200.0]	800.0 [600.0, 1200.0]	900.0 [600.0, 1200.0]	900.0 [600.0, 1200.0]	900.0 [550.0, 1200.0]	0.003	14.4
Total prime volume indexed to body surface area, median	1110.0 [1060.0, 1185.0]	1085.0 [1060.0, 1185.0]	1150.0 [1060.0, 1185.0]	1085.0 [1010.0, 1185.0]	1160.0 [1060.0, 1185.0]	0.001	57.8
Retrograde autologous priming	34,981 (85.8)	8877 (86.5)	8072 (86.8)	8445 (86.8)	9587 (83.4)	<0.001	0
Net prime volume (mL/m^2^) indexed to BSA	330.0 [277.8, 403.6]	321.3 [278.3, 394.9]	319.5 [268.8, 383.1]	334.1 [275.8, 406.2]	349.2 [288.4, 427.9]	<0.001	57.8
Acute normovolemic hemodilution	11,172 (27.4)	2688 (26.2)	2297 (24.7)	2692 (27.7)	3495 (30.4)	<0.001	0
Ultrafiltration							
Conventional ultrafiltration	10,118 (25.2)	2763 (27.1)	2261 (24.5)	2334 (24.2)	2760 (24.9)	<0.001	1.5
Post-cardiopulmonary bypass ultrafiltration	353 (2.6)	90 (2.4)	66 (2.1)	72 (2.2)	125 (3.4)	0.002	2.4
Ultrafiltration volume (non-CPB), median	300.0 [100.0, 500.0]	300.0 [200.0, 500.0]	300.0 [137.5, 500.0]	300.0 [0.0, 500.0]	300.0 [100.0, 500.0]	0.679	11.0
Ultrafiltration volume (CPB), median	1500.0 [900.0, 2800.0]	1600.0 [1000.0, 3000.0]	1500.0 [900.0, 3000.0]	1500.0 [800.0, 2700.0]	1500.0 [825.0, 2500.0]	<0.001	1.7
Cardiotomy suction	36,330 (89.7)	8689 (85.4)	8117 (87.7)	8835 (91.6)	10,689 (93.5)	<0.001	0.6
Autotransfusion	33,556 (82.8)	8262 (80.8)	7743 (83.6)	8102 (83.8)	9449 (83.2)	<0.001	0.6
Cell salvaged blood transfused, median	0.0 [0.0, 100.0]	0.00 [0.0, 225.0]	0.0 [0.0, 130.0]	0.0 [0.0, 89.0]	0.0 [0.0, 0.0]	<0.001	14.9
Augmented venous drainage						<0.001	0.7
None	14,455 (35.7)	4022 (39.6)	3361 (36.5)	3581 (37.0)	3491 (30.5)		
Vacuum	25,704 (63.5)	6143 (60.4)	5853 (63.5)	6079 (62.9)	7629 (66.7)		
Kinetic	337 (0.8)	3 (0.0)	4 (0.0)	8 (0.1)	322 (2.8)		
Total urine output on CPB (mL/m^2^), median	290.0 [170.0, 500.0]	285.0 [162.8, 485.0]	280.0 [160.0, 475.0]	284.5 [170.0, 500.0]	300.0 [175.0, 500.0]	<0.001	2.3

Continuous variables are expressed as median, [IQR], and categorical variables as count (%). Abbreviations: HCT, hematocrit; BSA, body surface area; CPB, cardiopulmonary bypass.

Anticoagulation monitoring was performed using activated clotting time and heparin concentration devices in 97.8% and 14.7% of all procedures, respectively, (Supplementary Table 3). Intraoperative viscoelastic testing was infrequently (25.6%) used, although increased over time (20.1% vs. 32.3%). While a ratio dose of heparin given during surgery was the most common (63.5%) method of calculating the protamine dose for anticoagulation reversal, its use diminished over time (68.9% vs. 52.4%) in part attributed to the increased use of heparin protamine titration (23.5% vs. 37.0%). The use of intraoperative insulin was reported in 80.8% of all procedures (Supplementary Table 4).

Cardiotomy suction was terminated following protamine administration in 70.2% of procedures, a practice which increased from 54.8% to 75.9% of procedures over time, Table 5. The overall rate of visible clot noted in the CPB circuit was 0.6%. The majority (99.6%) of procedures used a perfusion checklist. Transfer of care during the intraoperative period occurred in 10.5% of cases. The rate of any of the ten intraoperative adverse events decreased from 0.7% of cases to 0.5%.

**Table 5:** Patient safety.

	Overall	Year				*p*-value	Missing
		2019	2020	2021	2022		
Number of cases	40,777	10,261	9295	9731	11,490		
Timing of pump sucker termination						<0.001	7.6
Prior to, or at the initiation of, protamine delivery	26,447 (70.2)	5479 (54.8)	6034 (73.5)	6853 (77.9)	8081 (75.9)		
1%–25% of protamine given	2641 (7.0)	1393 (13.9)	431 (5.2)	382 (4.3)	435 (4.1)		
26%–50% of protamine given	6841 (18.2)	2340 (23.4)	1537 (18.7)	1333 (15.1)	1631 (15.3)		
>50% of protamine given	1730 (4.6)	788 (7.9)	213 (2.6)	234 (2.7)	495 (4.7)		
Evidence of visible clotting in the circuit	246 (0.6)	74 (0.7)	42 (0.5)	56 (0.6)	74 (0.7)	0.09	1.8
Perfusion checklist	40,146 (99.6)	10,039 (99.7)	9139 (99.7)	9603 (99.6)	11,365 (99.6)	0.971	1.2
Transfer of care during the intraoperative period	4213 (10.5)	968 (9.6)	1024 (11.2)	995 (10.3)	1226 (10.8)	0.003	1.4
Adverse event during the intraoperative period (y/n)	224 (0.6)	71 (0.7)	57 (0.6)	44 (0.5)	52 (0.5)	0.035	2.4

Continuous variables are expressed as median, [IQR], and categorical variables as count (%).

## Discussion

This first annual PERForm registry report provides important benchmarking information for cardiac surgical operations utilizing CPB. Since its inception, the PERForm registry has developed a robust infrastructure that supports the onboarding of new hospitals, the matching of institutional perfusion and surgical harvest files, the dissemination of quarterly benchmarking reports, and quality improvement. Given its growth, partnership with AmSECT, range of academic and community hospitals, as well as penetration outside of the state of Michigan, the registry now is prepared to provide benchmarking data to the wider cardiac surgical community through an annual report. Future reports will focus on specific practices, patterns of care, and associated outcomes.

This report adds to the literature in three important ways. First, to our knowledge, this report is among the first to use clinically informed data to describe discrete CPB practices. Second, this report provides contemporaneous data reflecting adherence to evidence-based guidelines and professionally based standards and guidelines, Supplementary Table 5. Third, this report is among the first to document the rate of intraoperative adverse events secondary to CPB procedures, including overall and annual trends in patient safety practices (Table 5). The findings derived from this report benefit from the increasing penetration of the PERForm registry across the United States (Supplementary Figure 1).

Clinical registry participation supports the assessment of care provided to adult cardiac surgical patients. Surveys have traditionally been used to establish benchmarks for cardiovascular perfusion, including establishing trends in practice patterns [14, 15], gaining consensus on essential clinical skills [16], and reporting adverse intraoperative incidents [17]. Unfortunately, this methodological approach is subject to bias, including recall bias (when asking a respondent to recall the number of instances of a particular adverse outcome) and survey bias (survey respondents versus non-respondents may differ in known and unknown ways). While the distribution of surveys provides the opportunity to amass large analytical datasets, investigators have reported variable survey response rates focused on both the cardiac surgical program (35%–100%) [14, 15] and perfusionist levels (52%–69%) [17–19]. As such, one of the distinct contributions of the present report is the establishment of benchmarks that derive from clinical registry data whose denominators are validated against institutional STS-ACSD harvest files. The validation against the STS-ACSD is important to minimize bias in data submitted by PERForm participants.

This first annual report highlights several emerging trends in CPB practices. The most recent 2021 STS/SCA/AmSECT/SABM patient blood management guidelines identified several evidence-based perfusion interventions, including retrograde autologous priming (RAP), reduced CPB priming volume, and acute normovolemic hemodilution (ANH) [20]. While the present report documents that 85.8% of procedures utilized RAP, its use decreased marginally over the study period (2019 vs. 2022: 86.5% vs. 83.4%, *p* < 0.001). An observational study of participating PERForm hospitals in 2014 found RAP usage in the setting of isolated CABG was 71.4%, suggesting a 21% increase over the last 7 years [21]. The median (IQR) net prime volume indexed to a patient’s body surface area increased from 321.3 mL/m^2^ (278.3–394.9) in 2019 to 349.2 mL/m^2^ (288.4%–429.9%) in 2022, *p* < 0.001. A previous PERForm analysis documented a median (IQR) indexed net prime volume of 378 mL/m^2^ for cases performed between July 2011 through December 2016 [22]. Despite evidence supporting their use [20], ANH (Level A evidence, rate: 25.4%) and viscoelastic testing (Level B–R evidence, rate: 22.8%) were not employed among the majority of procedures in the PERForm registry. Opportunities to enhance their use may be realized by leveraging local multidisciplinary workgroups, as ANH requires close collaboration between anesthesia and perfusion personnel to safely perform the sequestration process. While viscoelastic sampling may not be performed at the point of care among the majority of procedures within the present sample, interpreting the results, and guiding therapeutic decisions must be a coordinated approach between surgical team members [23].

Over the more than seven decades since the initial use of CPB by Dr. John Gibbon, Jr., there continue to be significant opportunities to advance the care and outcomes of patients undergoing cardiac surgery. Several initiatives have been undertaken by professional societies to address gaps in observed versus idealized outcomes, including but not limited to the creation and dissemination of evidence-based guidelines [20, 24–27] and professional consensus-based standards and guidelines [28]. Nonetheless, prior studies have identified significant gaps in translating evidence-based guidelines into practice [29]. For instance, a large international survey was conducted of cardiac surgical team members to evaluate the uptake of the 2007 STS blood management guidelines [29]. The 1402 returned surveys (32% response rate) represented 677 U.S. and 34 Canadian institutions. While the majority of perfusionists (67%) and anesthesiologists (78%) reported having read some and/or all the guidelines, institutional discussions were noted to have occurred by only 20% of respondents, with only 14% of respondents reporting the development of an institutional monitoring group. Some investigators have also leveraged registries to track the penetration of published evidence-based guidelines into practice efficiently [30], with derivative quality improvement initiatives used to address observed gaps in practice [31]. Lohbusch and colleagues recently reported findings from the analysis of a survey distributed to the chiefs of perfusion at 167 adult cardiac surgical programs located within AmSECT’s Zone IV covering 16 Atlantic states [15]. While receiving a 34.7% response rate, the investigators noted large-scale variability in the use of practices within AmSECT’s Standards and Guidelines. To our knowledge, this report is among the largest studies to leverage registry data to track trends in the dissemination of evidence-based guidelines, and the first registry-based study evaluating professionally based standards and guidelines.

The assessment of adverse CPB-related events traditionally has been undertaken through surveys [18, 19] and voluntary incident reporting systems [17, 32, 33]. Established in 1998, the Australia and New Zealand College of Perfusionists’ Perfusion Incident Reporting System (PIRS) is an incident reporting system within and outside of Oceania [17, 32]. More recently, Colligan and colleagues described the development and early findings derived from a North American incident and near-miss registry [33]. Designed as a federally designated Patient Safety Organization (PSO), the ORRUM PSO has recently partnered with AmSECT to provide professionally based patient safety work products. In both PIRS and ORRUM PSO, submitted reports are analyzed to derive key lessons learned. Uniquely, the present report documents events that are linked to clinically submitted registry data to derive rates for benchmarking and local quality improvement. Participants of the PERForm registry have access to online query tools to support further inquiry into submitted events and receive quarterly reports to facilitate benchmarking.

Institutional quality improvement (QI) programs aim to advance the safety and effectiveness of patient care by applying a systems approach for testing and implementing changes in day-to-day clinical practice [5, 34]. Unfortunately, such programs are often challenged by a lack of robust data collection and monitoring systems. Participation in multicenter clinical registries, including the PERForm and STS-ACSD, may facilitate both the assessment and improvement of care especially when grounded in a robust collaborative learning environment. A collaboration between the MSTCVS-QC and the Michigan Perfusion Society has resulted in several successful evidence-based, statewide perfusion-specific QI initiatives [22, 34]. A collaborative learning environment, whose foundation includes validated data and hospital center-level performance within the confines of quarterly collaborative meetings, has been instrumental to the success of this partnership. Further dissemination and expansion (e.g., including anesthesiologists) of this collaborative learning model is warranted to advance the interdisciplinary nature of CPB practices.

Several study-related limitations are worthy of discussion. While a large, multicenter experience, practice patterns noted in this report from 42 hospitals may have limited generalizability to other adult cardiac surgical programs. Although the degree of missing data is relatively low across most fields and data are audited across hospitals inferences derived from this report should be framed within the context of data quality. Last, this report intended to describe emerging trends in practice patterns; nonetheless, one cannot rule out the impact of unmeasured confounding given the observational nature of this study.

This first annual report of the PERForm registry seeks to provide important benchmarking information specific to the conduct of adult CPB. While the dissemination of this information is important, advancements in the delivery and outcomes of perfusion practices require local engagement through multidisciplinary work groups.

## Data Availability

Data use agreements restrict the distribution of raw study-related data files. Requests for summary statistics will be shared upon review and approval by the study team.

## Supplementary material

The supplementary material of this article is available at https://ject.edpsciences.org/10.1051/ject/2024006/olm *Supplementary Table 1*: Society of Thoracic Surgeons Adult Cardiac Surgery Registry procedure. *Supplementary Table 2*: Prime Constituents. *Supplementary Table 3*: Anticoagulation Management *Supplementary Table 4*: Glucose and Temperature Management. *Supplementary Table 5*: Data Elements Associated with AmSECT Evidence Based Clinical Guidelines and/or AmSECT Standards and Guidelines. *Supplementary Figure 1*: Trends in procedural count and hospital participation in the PERForm registry.
