# Supplementary material for: Perfusion Measures and Outcomes (PERForm) registry: First annual report
Source: J Extra Corpor Technol. 2024 Jun 18;56(2):55–64. doi: 10.1051/ject/2024006 (PMC11185137; doi:10.1051/ject/2024006)

Supplemental Material

Perfusion Measures and Outcomes (PERForm) Registry: First Annual Report

Table of Contents

Supplemental Table 1. Society of Thoracic Surgeons Adult Cardiac Surgery Registry procedure *3*

Supplemental Table 2. Prime Constituents. *4*

Supplemental Table 3. Anticoagulation Management. *6*

Supplemental Table 4. Glucose and Temperature Management. *7*

Supplemental Table 5. Data Elements Associated with AmSECT Evidence Based Clinical Guidelines and/or AmSECT Standards and Guidelines *8*

Supplemental Figure 1: Trends in procedural count and hospital participation in the PERForm registry *10*

Supplemental Table 1. Society of Thoracic Surgeons Adult Cardiac Surgery Registry procedure

	Other	CAB	MVR + CAB	AVR + CAB	MVR	MVr	AVR	AVR + MVR	MVr + CAB
Count	8,374	22,406	447	2,109	1,458	2,187	2,833	302	661

Abbreviations: CAB - Coronary Artery Bypass; MVR- Mitral Valve Replacement; AVR - Aortic valve Replacement; MVr - Mitral Valve Repair

Supplemental Table 2. Prime Constituents.

		Year				p-value	Missing
	**Overall**	**2019**	**2020**	**2021**	**2022**		
**Number of cases**	40777	10261	9295	9731	11490		
Heparin volume	10.0 [5.0, 10.0]	10.0 [10.0, 10.0]	10.0 [10.0, 10.0]	10.0 [5.0, 10.0]	10.0 [5.0, 10.0]	<0.001	1.5
Heparin Dose, units	10000.0 [10000.0, 10000.0]	10000.0 [10000.0, 10000.0]	10000.0 [10000.0, 10000.0]	10000.0 [5000.0, 10000.0]	10000.0 [5000.0, 10000.0]	<0.001	1.2
**Albumin 25% Use**	19104 (46.8)	4740 (46.2)	3972 (42.7)	4522 (46.5)	5870 (51.1)	<0.001	0
**Albumin 25% Volume**						<0.001	10.6
0	17336 (47.6)	4001 (45.8)	4092 (50.7)	4329 (48.9)	4914 (45.6)		
50	12654 (34.7)	3316 (37.9)	2732 (33.9)	2905 (32.8)	3701 (34.3)		
70	13 (0.0)	8 (0.1)	5 (0.1)	0 (0.0)	0 (0.0)		
100	3583 (9.8)	772 (8.8)	817 (10.1)	915 (10.3)	1079 (10.0)		
150	1345 (3.7)	413 (4.7)	199 (2.5)	197 (2.2)	536 (5.0)		
200	648 (1.8)	184 (2.1)	158 (2.0)	161 (1.8)	145 (1.3)		
	290 (0.7)	164 (1.6)	91 (1.0)	17 (0.2)	18 (0.2)		
**Albumin 5% Volume**						<0.001	38.1
0	24950 (98.9)	5355 (97.0)	5614 (98.4)	6461 (99.7)	7520 (99.8)		
50	7 (0.0)	4 (0.1)	1 (0.0)	0 (0.0)	2 (0.0)		
670	1 (0.0)	1 (0.0)	0 (0.0)	0 (0.0)	0 (0.0)		
**Starch Solution Volume**						0.482	38.4
0	25136 (100.0)	5441 (100.0)	5682 (100.0)	6477 (100.0)	7536 (100.0)		
50	2 (0.0)	0 (0.0)	1 (0.0)	0 (0.0)	1 (0.0)		
100	1 (0.0)	1 (0.0)	0 (0.0)	0 (0.0)	0 (0.0)		
**Sodium Bicarbonate Dose, meq**	30.0 [0.0, 50.0]	50.0 [0.0, 50.0]	50.0 [0.0, 50.0]	30.0 [0.0, 50.0]	25.0 [0.0, 50.0]	<0.001	14.7
**Sodium Bicarbonate Volume**	40.0 [0.0, 50.0]	50.0 [0.0, 50.0]	50.0 [0.0, 50.0]	30.0 [0.0, 50.0]	25.0 [0.0, 50.0]	<0.001	14.7
**Mannitol Dose, grams**	12.5 [12.00, 12.5]	12.5 [12.5, 25.0]	12.5 [12.0, 12.5]	12.5 [12.0, 12.5]	12.5 [12.0, 12.5]	<0.001	16.9
**Mannitol Volume**	50.0 [50.0, 62.8]	50.0 [50.0, 100.0]	50.0 [50.0, 60.0]	50.0 [50.0, 60.0]	50.0 [50.0, 50.0]	<0.001	16.9
**Balanced Electrolyte Solution Volume**	1000.0 [800.0, 1200.0]	1000.0 [800.0, 1200.0]	1000.0 [800.0, 1200.0]	1000.0 [800.0, 1200.0]	1000.0 [800.0, 1100.0]	<0.001	1.6
**Hartmanns Solution Volume**						0.41	44.6
0	22571 (100.0)	5443 (100.0)	5681 (100.0)	6453 (100.0)	4994 (100.0)		
1100	1 (0.0)	1 (0.0)	0 (0.0)	0 (0.0)	0 (0.0)		
1200	1 (0.0)	0 (0.0)	1 (0.0)	0 (0.0)	0 (0.0)		

**Lactated Ringers Volume**						<0.001	38.2
0	24602 (97.7)	5446 (99.8)	5681 (99.6)	6480 (100.0)	6995 (92.8)		
200	1 (0.0)	0 (0.0)	1 (0.0)	0 (0.0)	0 (0.0)		
600	1 (0.0)	0 (0.0)	0 (0.0)	0 (0.0)	1 (0.0)		
1000	31 (0.1)	10 (0.2)	19 (0.3)	2 (0.0)	0 (0.0)		
1100	115 (0.5)	0 (0.0)	0 (0.0)	0 (0.0)	115 (1.5)		
1200	1 (0.0)	0 (0.0)	1 (0.0)	0 (0.0)	0 (0.0)		
1250	1 (0.0)	0 (0.0)	0 (0.0)	0 (0.0)	1 (0.0)		
1300	426 (1.7)	0 (0.0)	0 (0.0)	0 (0.0)	426 (5.6)		
1400	1 (0.0)	0 (0.0)	0 (0.0)	0 (0.0)	1 (0.0)		
2000	1 (0.0)	0 (0.0)	0 (0.0)	0 (0.0)	1 (0.0)		
**Saline Volume**						<0.001	36.9
0	24939 (96.9)	5315 (95.0)	5672 (96.6)	6418 (96.6)	7534 (98.7)		
200	21 (0.1)	21 (0.4)	0 (0.0)	0 (0.0)	0 (0.0)		
250	1 (0.0)	1 (0.0)	0 (0.0)	0 (0.0)	0 (0.0)		
300	10 (0.0)	10 (0.2)	0 (0.0)	0 (0.0)	0 (0.0)		
500	5 (0.0)	2 (0.0)	1 (0.0)	2 (0.0)	0 (0.0)		
750	1 (0.0)	1 (0.0)	0 (0.0)	0 (0.0)	0 (0.0)		
1000	10 (0.0)	3 (0.1)	4 (0.1)	3 (0.0)	0 (0.0)		
1200	27 (0.1)	24 (0.4)	2 (0.0)	0 (0.0)	1 (0.0)		

Continuous variables are expressed as median, [IQR], and categorical variables as count (%).

Supplemental Table 3. Anticoagulation Management.

		Year					
	**Overall**	**2019**	**2020**	**2021**	**2022**	**p-value**	**Missing**
**Number of cases**	40777	10261	9295	9731	11490		
**Method for Monitoring Anticoagulation**							
ACT	39898 (97.8)	10103 (98.5)	9150 (98.4)	9569 (98.3)	11076 (96.4)	<0.001	0
Heparin concentration (e.g., HMS, heparin-protamine titration)	5999 (14.7)	1270 (12.4)	1234 (13.3)	1497 (15.4)	1998 (17.4)	<0.001	0.2
PT/PTT	1 (0.0)	1 (0.0)	0 (0.0)	0 (0.0)	0 (0.0)	<0.001	0.2
**Viscoelastic Testing Used**	10336 (25.6)	2051 (20.1)	1951 (21.0)	2689 (27.9)	3645 (32.3)	<0.001	0.9
**Heparin dose response (HDR) for determining initial heparin dose**	10315 (25.5)	2050 (20.1)	1951 (21.0)	2689 (27.9)	3625 (32.2)	<0.001	0.9
**Initial Heparin Dose given, median**	31000.0 [26000.0, 38000.0]	30000.0 [27000.0, 38000.0]	32000.0 [27000.0, 40000.0]	31000.0 [26000.0, 40000.0]	30000.0 [25000.0, 37000.0]	<0.001	1.3
**Method for Calculating Initial Protamine Dose**						<0.001	0.8
Protamine not given	9 (0.0)	0 (0.0)	5 (0.1)	3 (0.0)	1 (0.0)		
Fixed dose	2823 (7.0)	729 (7.1)	540 (5.8)	527 (5.5)	1027 (9.1)		
Heparin protamine titration (HPT)	11516 (28.5)	2404 (23.5)	2074 (22.4)	2855 (29.6)	4183 (37.0)		
Ratio dose of heparin given	25685 (63.5)	7046 (68.9)	6592 (71.1)	6125 (63.5)	5922 (52.4)		
Other	410 (1.0)	44 (0.4)	60 (0.6)	138 (1.4)	168 (1.5)		
Total Protamine Dose, median	300.0 [250.0, 400.0]	300.0 [250.0, 400.0]	300.0 [250.0, 400.0]	300.0 [250.0, 350.0]	275.0 [250.0, 350.0]	<0.001	2.5

Continuous variables are expressed as median, [IQR], and categorical variables as count (%). Abbreviations: ACT, activated clotting time; HMS, heparin management system; PT, prothrombin time; PTT, partial thromboplastin time

Supplemental Table 4. Glucose and Temperature Management.

		Year				p-value	Missing
	**Overall**	**2019**	**2020**	**2021**	**2022**		
**Number of cases**	40777	10261	9295	9731	11490		
**Glucose**							
Highest intra-operative glucose	169.0 [148.0, 196.0]	169.0 [147.0, 198.0]	170.0 [149.0, 196.0]	168.0 [147.0, 195.0]	169.0 [148.0, 196.0]	0.007	2
Intraoperative Insulin drip	30976 (80.8)	7585 (77.6)	7186 (81.4)	7574 (82.6)	8631 (81.8)	<0.001	6
**Temperature**							
Lowest core	34.3 [33.5, 35.0]	34.2 [33.3, 35.0]	34.3 [33.6, 35.0]	34.3 [33.7, 35.0]	34.2 [33.5, 35.0]	<0.001	0.6
Maximum arterial line	37.0 [36.8, 37.0]	37.0 [36.8, 37.0]	37.0 [36.8, 37.0]	37.0 [36.8, 37.0]	37.0 [36.7, 37.0]	<0.001	0.5

Continuous variables are expressed as median, [IQR], and categorical variables as count (%).

Supplemental Table 5. Data Elements Associated with AmSECT Evidence Based Clinical Guidelines and/or AmSECT Standards and Guidelines

**Disposable & Monitoring Equipment**	
Bio Coating	SIRS (Landis, et al) [Suppl Citation#1]
Arterial Pump Device	SIRS (as part of MiECC) (Landis, et al) [Suppl Citation#1]
Perfusion Electronic Medical Record	AmSECT Standards and Guidelines for Perfusion Practice[28]
Cerebral Oximetry Device Usage	AmSECT Standards and Guidelines for Perfusion Practice[28]
Inline Blood Gas Trending Device	AmSECT Standards and Guidelines for Perfusion Practice[28]
**Cardioplegia Details**	
Use of Cardioplegia	SIRS (Landis, et al) [Suppl Citation#1]
Cardioplegia Regime	N/A
Cardioplegia Solution (Category)	N/A
Induction Routes	N/A
Maintenance Route	N/A
Hot shot used	N/A
**Blood Product Utilization and Fluid Management**	
Blood Product Utilization	Blood (Tibi, et al)[20]
Hematocrit Values	Blood (Tibi, et al)[20]
HCT prior to first and second intraoperative transfusion	Blood (Tibi, et al)[20]
Prime Volume (including retrograde autologous priming)	Blood (Tibi, et al)[20], SIRS (as part of MiECC) (Landis, et al) [Suppl Citation#1]
Acute Normovolemic Hemodilution	Blood (Tibi, et al)[20]
Ultrafiltration	Blood (Tibi, et al)[20], Renal (Brown, et al)[24]
Autotransfusion	Blood (Tibi, et al)[20]
Cardiotomy Suction	Renal (Brown et al)[24], SIRS (as part of MiECC) (Landis, et al) [Suppl Citation#1]
Augmented Venous Drainage	SIRS (as part of MiECC) [Suppl Citation#1]
Total Urine Output	Renal (Brown, et al)[24]
**Prime Constituents**	
Albumin 5%/25% Volume	Blood (Tibi, et al)[20]
Starch Solution	Blood (Tibi, et al)[20]
Sodium Bicarbonate Dose/Volume	N/A
Mannitol grams/volume	N/A
Balanced Electrolyte Solution Volume	N/A
Hartmann's Solution Volume	N/A
Lactated Ringers Volume	N/A
0.9% Saline Volume	N/A

Anticoagulation Management	
Initial Heparin Dose Given	Anticoagulation (Shore-Lesserson, et al)[25]
Heparin Dose/Volume	N/A
Method for Monitoring Anticoagulation	Anticoagulation (Shore-Lesserson, et al)[25]
Viscoelastic Testing Used	Anticoagulation (Shore-Lesserson, et al)[25], Blood (Tibi, et al)[20]
Heparin Dose Response for Determining Initial Heparin dose	Anticoagulation (Shore-Lesserson, et al)[25]
Method for Calculating Initial Protamine Dose	Anticoagulation (Shore-Lesserson, et al)[25]
**Glucose and Temperature Management**	
Glucose (Highest)	Renal (Brown, et al)[24]
Temperature (Lowest/Highest)	Temperature (Engelman, et al) [26], Renal (Brown, et al)[24]
**Patient Safety**	
Timing of Pump Sucker Termination	AmSECT Standards and Guidelines for Perfusion Practice[28]
Evidence of Visible Clotting in the Circuit	Anticoagulation (Shore-Lesserson, et al)[25]
Perfusion Checklist	AmSECT Standards and Guidelines for Perfusion Practice[28]
Transfer of Care During the Intraoperative Period	AmSECT Standards and Guidelines for Perfusion Practice[28]
Adverse Event During the Intraoperative Period	AmSECT Standards and Guidelines for Perfusion Practice[28]

Abbreviations: SIRS - systemic inflammatory response syndrome; AmSECT – American Society of ExtraCorporeal Technology

Suppl Citation#1: Landis RC, Brown JR, Fitzgerald D, et al. Attenuating the Systemic Inflammatory Response to Adult Cardiopulmonary Bypass: A Critical Review of the Evidence Base. J Extra Corpor Technol. 2014;46(3):197-211.

Supplemental Figure 1: Trends in procedural count and hospital participation in the PERForm registry

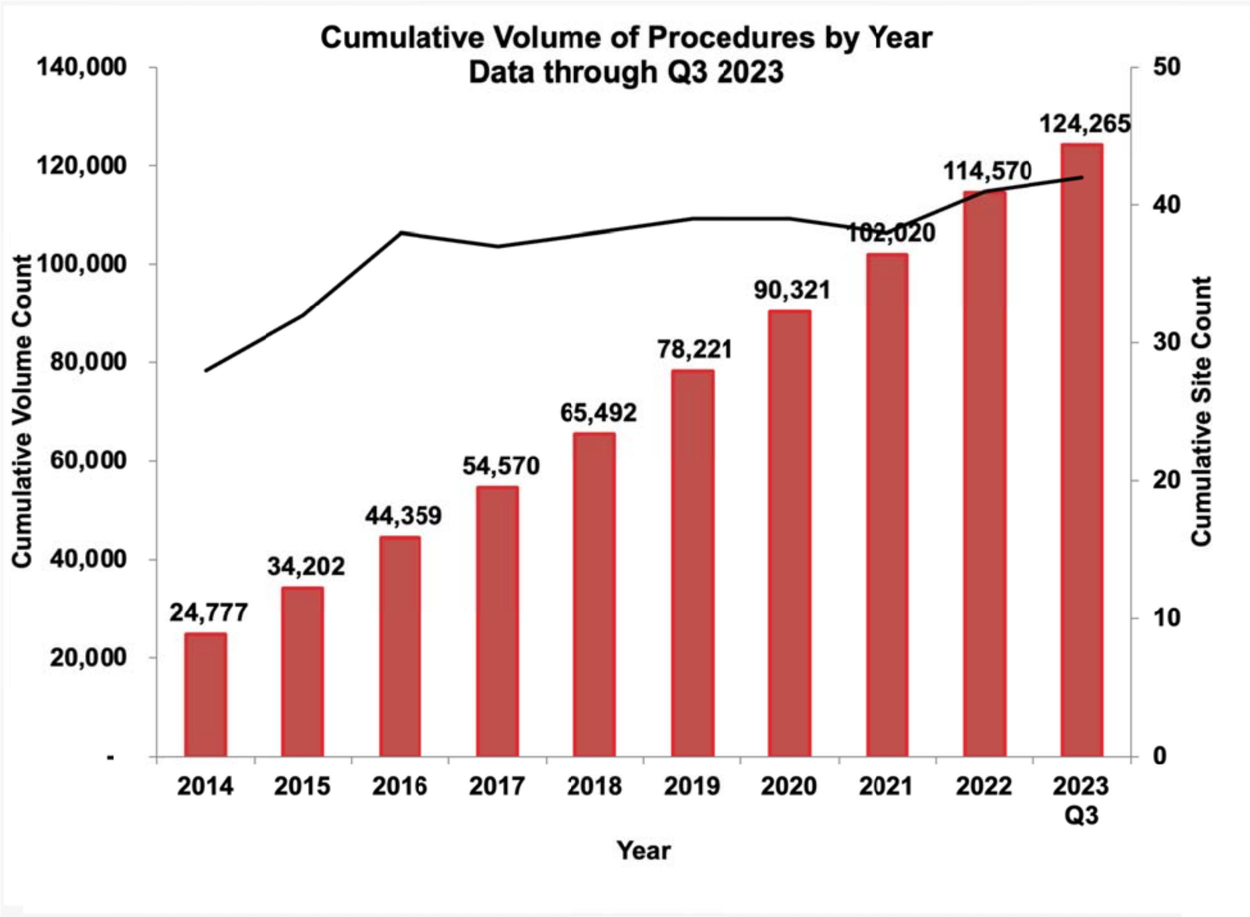

Supplement: Supplementary file 1 — Supplementary Table 1: Society of Thoracic Surgeons Adult Cardiac Surgery Registry procedure. Supplementary Table 2: Prime Constituents. Supplementary Table 3: Anticoagulation Management Supplementary Table 4: Glucose and Temperature Management. Supplementary Table 5: Data Elements Associated with AmSECT Evidence Based Clinical Guidelines and/or AmSECT Standards and Guidelines. Supplementary Figure 1: Trends in procedural count and hospital participation in the PERForm registry. [file ject-56-55-s1.pdf]
